# Exploring Technology- and Sensor-Driven Trends in Education: A Natural-Language-Processing-Enhanced Bibliometrics Study †

**DOI:** 10.3390/s23239303

**Published:** 2023-11-21

**Authors:** Manuel J. Gomez, José A. Ruipérez-Valiente, Félix J. García Clemente

**Affiliations:** Faculty of Computer Science, University of Murcia, 30100 Murcia, Spainfgarcia@um.es (F.J.G.C.)

**Keywords:** technology-enhanced learning, sensor-based learning, bibliometrics, natural language processing, social network analysis

## Abstract

Over the last decade, there has been a large amount of research on technology-enhanced learning (TEL), including the exploration of sensor-based technologies. This research area has seen significant contributions from various conferences, including the European Conference on Technology-Enhanced Learning (EC-TEL). In this research, we present a comprehensive analysis that aims to identify and understand the evolving topics in the TEL area and their implications in defining the future of education. To achieve this, we use a novel methodology that combines a text-analytics-driven topic analysis and a social network analysis following an open science approach. We collected a comprehensive corpus of 477 papers from the last decade of the EC-TEL conference (including full and short papers), parsed them automatically, and used the extracted text to find the main topics and collaborative networks across papers. Our analysis focused on the following three main objectives: (1) Discovering the main topics of the conference based on paper keywords and topic modeling using the full text of the manuscripts. (2) Discovering the evolution of said topics over the last ten years of the conference. (3) Discovering how papers and authors from the conference have interacted over the years from a network perspective. Specifically, we used *Python* and *PdfToText* library to parse and extract the text and author keywords from the corpus. Moreover, we employed *Gensim* library Latent Dirichlet Allocation (LDA) topic modeling to discover the primary topics from the last decade. Finally, *Gephi* and *Networkx* libraries were used to create co-authorship and citation networks. Our findings provide valuable insights into the latest trends and developments in educational technology, underlining the critical role of sensor-driven technologies in leading innovation and shaping the future of this area.

## 1. Introduction

The term technology-enhanced learning (TEL) is used to describe the application of information and communication technologies to teaching and learning [1]. With the continuous advancements in technology, its impact on educational environments has been significant, including the potential integration of sensor technologies. Sensor-driven applications in education, such as augmented reality (AR) and virtual reality (VR) platforms, mobile learning devices, and other integrated systems, have been at the forefront of educational innovation. Despite certain limitations that prevent the broader implementation of technology in education, such as economic barriers [2], interest and research in this area have been steadily increasing over the years. This increase serves as an excellent motivation to analyze the current trends in educational technology (EdTech) and identify emerging patterns that potentially incorporate sensor-driven solutions.

However, conducting a comprehensive analysis of a rapidly expanding body of literature presents significant challenges. Traditional approaches, such as systematic reviews, scoping reviews, or even meta-reviews of multiple review papers [3,4], are often time-consuming and may not fully capture the dynamic nature of the field. Analyzing and understanding research methods involves carefully studying the existing literature. Often, this is accomplished by individuals or groups focusing on a specific set of publications that is manually reviewed [5]. As the TEL literature continues to grow, there is a need for innovative methodologies that can quickly and effectively analyze large amounts of research, capturing valuable insights that may otherwise remain hidden within the literature.

Several research areas could alleviate the workload required for a review. Natural Language Processing (NLP), for example, aims to accomplish human-like language processing, being able to translate texts, answer questions about their content, or even draw inferences from the text [6]. Other areas can provide us with even more information than a manual review very quickly, such as the area of bibliometrics (i.e., the application of mathematics and statistical methods to books and other media of communication [7]) or network analysis, which can evidence relationships between authors and papers. To address these challenges and capture the rich insights afforded by the intersection of sensors and EdTech, we use a novel open science methodology that is quick and scalable.

The integration of new technologies in education, including sensors, and the fast evolving way in which we generate, process, and revise information are bound to have a major impact on how we teach and learn [8]. Technology-enhanced learning has revolutionized traditional educational practices by leveraging innovative tools and applications to enhance teaching and learning experiences [9,10]. AR and VR simulations transport students to immersive virtual environments, while mobile devices equipped with various sensors (e.g., gyroscopes, accelerometers) allow for personalized learning experiences, adapting to individual preferences and movements. Additionally, Internet of Things (IoT)-integrated systems enable the collection of real-time data in educational environments. In fact, IoT technology has facilitated the consolidation of educational resources in recent years, creating scalable, media-content-rich repositories that can be further analyzed for valuable insights [11]. Through the use of technology and sensors, teachers can create dynamic learning spaces that adjust to each student’s needs and encourage active participation. As education continues to advance, incorporating technology and sensors opens doors to a future where learning becomes more interactive and customized to suit individual learner requirements. We believe this is a genuine motivation for examining and discovering the main topics in EdTech and its evolution during the past ten years.

In this research, we aim to explore and discover the main topics in the field of technology-enhanced learning (TEL) over the past ten years. With this purpose in mind, we propose an open-science approach that combines Natural Language Processing (NLP), topic modeling, and social network analysis. Firstly, we offer insights into the main topics by analyzing paper keywords along with topic modeling using the full-text manuscripts. Secondly, we investigate the evolution of these topics over the last decade, providing a comprehensive view of the shifting trends in the EdTech area. Finally, we examine author and paper interactions by analyzing co-authorship and references between manuscripts, revealing the collaborative dynamics in the field. In this way, our research contributes to the understanding of the EdTech area while emphasizing the critical role of sensor-driven technologies in shaping the future of education. Specifically, we have the following research questions:

1. What are the main topics of EC-TEL based on keywords and topic modeling using the full text of manuscripts from the last ten years?
2. What has been the evolution of said topics over the last ten years before the conference?
3. How have papers and authors interacted over the last ten years before the conference?

The rest of the paper is organized as follows. Section 2 reviews the background literature on bibliometrics, social network analysis, and trends in EdTech. Section 3 describes the entire methodology followed to conduct the research, and Section 4 presents the results of the synthesis and analysis. Then, we summarize the paper with a discussion in Section 5, and the conclusions and future work are presented in Section 6.

## 2. Related Work

### 2.1. Bibliometrics

The term “bibliometrics” was first defined, to the best of our knowledge, in the *Journal of Documentation* in 1969 [12]. Since then, multiple definitions have surfaced. For instance, it has been described as “the application of mathematics and statistical methods to books and other media of communication”, or “quantitative analyses of the bibliographic features of a body of literature” [13]. In addition, the term “scientometrics” is broader, involving “all quantitative aspects of the science of science.” As a result, there is significant overlap between bibliometrics and scientometrics [13,14].

We have found several studies in this area that aimed to analyze trends in certain fields [15,16,17]. For example, in a study by Gao et al. [15], the authors aimed to investigate the current state, popular topics, and future possibilities in the field of e-waste (electronic waste). They collected data from the Web of Science Core Collection and used different tools (e.g., *CiteSpace V*, *Histcite*) to analyze information from various research papers. Their analyses included aspects such as the types of documents and publication languages, yearly publication trends and projections, authors and co-cited authors, and countries and institutions involved, among other factors. Moreover, the authors of [17] used bibliometrics to analyze trends in blockchain technology research, retrieving 801 papers from Scopus and calculating insightful measures such as author productivity based on the number of papers published by each author.

### 2.2. Natural Language Processing

Another area that is related to our work is Natural Language Processing (NLP). NLP is a field of study and practical implementation that studies how computers can comprehend and manipulate natural language text or speech for useful purposes [18]. To offer a more in-depth insight into the concept, Elizabeth D. Liddy [6] provides an expanded description: “Natural Language Processing is a theoretically motivated range of computational techniques for analyzing and representing naturally occurring texts at one or more levels of linguistic analysis to achieve human-like language processing for a range of tasks or applications.” There have been some studies that have previously applied Natural Language Processing (NLP) on full-text manuscripts. In [19], the Association for Computational Linguistics (ACL) and Language Resources and Evaluation Conference (LREC) proceedings were analyzed by using a tool for NLP analysis, with the goal of inferring topic trends and research communities. Moreover, in [20], the authors applied topic modeling with full-text and abstract-only manuscripts to compare both models. The work in [21] goes a step further, and used the text that accompanies citations in scientific articles, along with supervised methods, to determine the purpose (i.e., author intention) and the polarity (i.e., author sentiment) of a reference. We have also found previous studies that analyzed trends in the EdTech area [22,23,24]. For example, the authors of [23] used text mining in e-learning publications that were grouped into groups and clusters based on an abstract analysis. In [24], the authors analyzed the contributions over five years to EC-TEL (from 2006 to 2010) and identified prolific authors, successful co-author networks, and the most cited publications.

Moreover, there are studies that have conducted topic trend analyses in the literature. In fact, Xin Li and Lei Lei [25] conducted a bibliometric analysis of topic modeling studies between 2000 and 2017, finding that LDA, social networks, and text analysis are the topics with an increasing popularity. We can find several examples of this type of study in previous research. For instance, the authors of [26] performed an extensive literature search on SARS-CoV-2 and COVID-19 publications, analyzing abstracts from 269,186 publications and identifying 10 topics. Chen Xieling et al. [27] conducted a topic trend analysis by using structural topic modeling based on 3900 learning analytics articles published during a decade. For the analysis of research topics, terms were extracted from titles and abstracts since they represent the main content of articles and have been commonly adopted to analyze research topics. Furthermore, Gurcan et al. [28] gathered metadata from 1925 peer-reviewed journal articles published between 2000 and 2019. They used abstracts from these papers to identify trends in the field using an LDA model. The study identified 16 different topics, highlighting key themes such as “MOOC”, “learning assessment”, and “e-learning systems”, which were consistently prevalent in the research literature. We found several additional studies addressing topic trend analyses in the scientific literature [29,30]. While these studies are usually focused on analyzing abstracts or only using text-based approaches, our research goes beyond the existing research by combining NLP techniques with a social network analysis, resulting in a more comprehensive and in-depth exploration of topic trends.

### 2.3. Social Network Analysis

Furthermore, network analysis is gaining attention as a general methodology for understanding complex patterns of interactions. It focuses on individuals or entities linked through various relationships, whether direct or indirect. Social network analysis has found applications in exploring family ties, social progress, academic citations, corporate influence, global trade dynamics, class hierarchies, and numerous other domains [31]. We found some works that have previously used social networks to analyze research papers. The researchers in [32] collected information from articles related to information sciences using databases like the *CSA Sociological Abstracts Database* (SA), *Medline Advanced*, and *PsycINFO*. Using these data, they created a social network of co-authorships to discover connections between authors who collaborated on papers. They applied various calculations, including density, degree centrality, and closeness, to measure the interactions within the network. Furthermore, the work in [24] built a co-authorship network using EC-TEL papers from 2006 to 2010, revealing a very fragmented community that is only weakly connected. Although this work did not build a citation network, it used the metadata to discover the most cited papers from the conference, finding that the most cited paper that has been published within EC-TEL is on MACE, published by Stefaner et al. in 2007 [33].

The possibility of enriching the indexed metadata with the full-text processing of papers offers a new research direction. The main problems arise from the organization and structure of the natural language, the extraction of information, and its representation [34]. Following our methodology, included in the research topic of “NLP-enhanced bibliometrics”, we aim to provide an easy and scalable way to analyze trends, going beyond the state of the art using full-text manuscripts combined with metadata. In addition, we use social network analysis to build two types of social networks: a co-authorship network and a citation network.

Finally, we can also highlight the main significance of our current work compared to our previous study [35], where we also aimed to provide an overview of the last ten years in the field. In the current research, we have made significant improvements in several aspects. Firstly, we have updated the corpus of papers, collecting manuscripts from the extended timeframe of 2013 to 2022. Unlike the original work, which relied on paper keywords to identify trends, our present study uses a novel approach by parsing the full text of the manuscripts and utilizing it to conduct a topic modeling analysis. This methodology has proven to yield more accurate and comprehensive results compared to only using paper abstracts [20]. Moreover, we introduced an additional layer of analysis by incorporating a social network analysis to explore interactions among authors and papers within the TEL community. Our work not only expands the temporal scope of the analysis, but also incorporates new stages in the methodology by combining full-text topic modeling and a social network analysis. Moreover, in order to make our study completely reproducible, we followed an open science approach and made the code and dataset accessible via OSF [36]. By sharing our code and dataset, we enable other researchers to validate our findings and replicate our methodology. This transparency not only allows for a greater confidence in the reliability of our results, but also encourages collaboration and further advancements built upon our work.

## 3. Methodology

**Figure 1:**
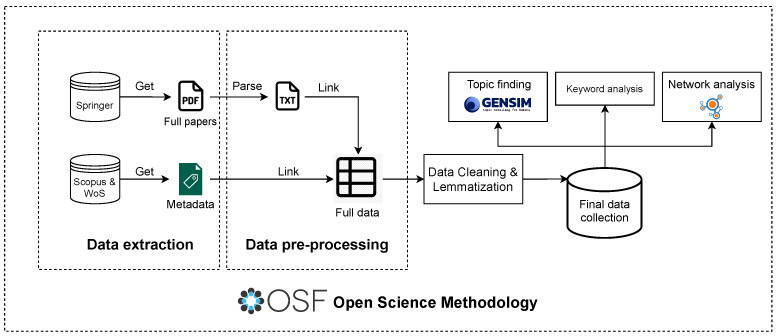
Complete methodology followed to conduct the research.

The European Conference on Technology-Enhanced Learning (EC-TEL) has become the primary EdTech conference in Europe and one of the world’s leading conferences, with a significant history of 18 years. We consider that this platform embraces research within the EdTech area from several different communities, including those exploring the potential of sensor-driven solutions, and is very representative of the trends in this area [24,37]. For that reason, we chose the EC-TEL to analyze and discover the main topics in EdTech and its evolution during the past ten years. To conduct the research, we divided our work in different stages: (A) pre-registration of the project, (B) data extraction, (C) data pre-processing, (D) data cleaning and lemmatization, (E) final data collection description, (F) NLP analysis, (G) network analysis, and (H) reproducible open data and analyses. The entire methodology process is represented in Figure 1. Now, let us present each part of the methodology in detail.

### 3.1. Pre-Registration of the Project

In this initial stage, we pre-registered our work as follows:

- We created an Open Science Framework (OSF) project [36].
- We pre-registered the research questions and methodology of the project.

### 3.2. Data Extraction

The first step in our analysis was to obtain all the data necessary to begin the research. These data included the full manuscripts of the research conference papers (full and short papers) over the last ten years, and the metadata (title, keywords, source, publication year…) of each paper downloaded. Hence, we excluded demo and poster papers in our review. Focusing on the most recent decade, our aim is to provide valuable insights into the latest advancements and emerging topics in the area.

On the one hand, to download each paper from the last decade, we used the Springer Link database [38]. On the other hand, we used two different databases to obtain each paper’s metadata: Scopus and Web of Science.

- Scopus [39] offers an extensive abstract and citation database combined with enriched data and linked scholarly content spanning various fields. Across the globe, Scopus is widely used by over 5000 academic, government, and corporate institutions, and plays a key role in supporting the research intelligence portfolio.
- Web of Science [40] is a valuable compilation of citation indexes that highlights the connections between scholarly research articles from prominent journals, books, and proceedings in different fields such as sciences, social sciences, and arts and humanities. It also serves as the basis for the journal impact metrics presented in Journal Citation Reports and the institutional performance metrics provided by InCites.

Using these two databases, we gathered different CSV and XLS files containing the full metadata required for the analysis. However, we could not locate the metadata corresponding to the papers from the EC-TEL 2015 edition. As a result, we manually added the necessary metadata for these papers to include them in our analysis.

### 3.3. Data Pre-Processing

In this stage, we processed the raw data acquired from the databases for subsequent analysis. The initial aspect of this stage involved converting each PDF file into a plain text (TXT) format. To accomplish this, we explored various Python libraries capable of performing this task. The selection of the most suitable library was based on the following steps:

1. **Search**. We explored five different libraries: *slate*, *pdfMiner*, *pdfPlumber*, *pyPdf*, and *PdfToText*.
2. **Parsing evaluation**. We assessed each library’s ability to successfully parse the PDF files.
3. **Manual text review**. We manually compared some TXT files with their corresponding raw PDF files to evaluate the quality of parsing.

Some libraries encountered difficulties parsing specific PDF files, while others resulted in empty TXT files after parsing. Finally, *PdfToText* emerged as the most reliable library, successfully parsing 100% of the papers while maintaining a high fidelity.

The following step included linking the plain text of each paper to its respective metadata. This was accomplished using Python for an automated process, merging the entire manuscript and its metadata into a unified data structure. This integration was achieved by analyzing the initial sentence of each paper’s full text and comparing it against the paper title.

### 3.4. Data Cleaning and Lemmatization

After gathering and pre-processing all the collected data, the next step involved cleaning the full text of each paper. In this process, we kept only the paper’s main body, including its abstract, while excluding the title, authors’ names, and references from the full text. Afterwards, further cleaning actions were executed to eliminate unnecessary elements such as URLs, numerical values, and excessive spaces. To prepare for later NLP techniques, a set of “stop words” was defined (words that will not be considered in the text analysis). In addition to the default set of stop words, we augmented the list based on a review of our data. Common terms such as “et”, “al”, “abstract”, “table”, or “figure” appear in almost every document but do not provide helpful information for the analysis.

With most of the cleaning process completed, we proceeded to treat each paper as a distinct “document”. Once the full text was cleaned, we performed lemmatization using the *pywsd* library. Lemmatization involves transforming a word into its base form, and in *pywsd*, this process is conducted as follows:

1. The string is tokenized, breaking it into individual tokens (words).
2. A Part-Of-Speech (POS) tagger assigns a POS tag (such as adverb, noun, adjective) to each word.
3. The lemmatizer is called with the token and its corresponding POS tag to obtain the word’s base form.

One of the key stages is assigning a POS tag to each word because this tag will remove language ambiguities. For example, if we want to lemmatize the sentence “The student was learning”, the correct result would be “the student be learn”. However, if we want to lemmatize the sentence “learning is good”, the result should be “learning be good”. This is because the word learning acts as a noun in the second sentence and as a verb in the first one. The POS tag solves this ambiguity and allows the lemmatizer to address these ambiguities correctly.

### 3.5. Final Data Collection Description

**Figure 2:**
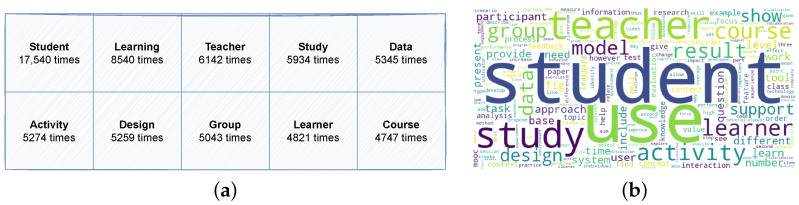
Initial exploration of the entire collection. (**a**) Most frequent words in the data collection. (**b**) Most representative words in the data collection.

The final data collection contains a total of 477 documents: 49 documents corresponding to 2013, 45 to 2014, 46 to 2015, 49 to 2016, 47 to 2017, 42 to 2018, 41 to 2019, 49 to 2020, 49 to 2021, and 60 to 2022. The overall corpus comprises 1,878,763 words, with 67,598 of these being unique. In terms of keywords, the entire dataset encompasses 2072 keywords distributed across 477 papers, with an average of 4.35 keywords per paper. Notably, 26 papers (5.5%) did not provide any keywords. In Figure 2a, we can see the ten most frequently occurring words in our document collection, along with the respective frequency of each word’s appearance. Then, in Figure 2b, we can observe the application of a word cloud model to our dataset. In both models, we can see common words including “student”, “use”, “teacher”, “data”, “group”, and “activity”. These findings align with our expectations, as the predominant and significant words revolve around the themes of learning and technology.

### 3.6. NLP Analysis

To discover the primary topics of the EC-TEL from the last decade, we employed Latent Dirichlet Allocation (LDA) topic modeling on our dataset. Prior research [20] has investigated two options for topic modeling on research papers: using the full text or solely the abstract. It was concluded that utilizing the full text yields a higher number of topics with greater coherence, consequently leading to improved results. Thus, we adopted the use of full manuscripts for our analysis, employing the *gensim* library and its *ldaMallet* model. The *ldaMallet* model applies an optimized Gibbs sampling algorithm for LDA [41].

There are multiple metrics for evaluating the optimal number of topics. Recent studies have revealed a lack of strong correlation between the classic predictive likelihood metric (or perplexity) and human judgment, occasionally even indicating a slight negative correlation [42]. This discrepancy has led to many studies focused on the development of topic coherence measures. In our study, we utilize two of these coherence measures: $C_{v}$ and $C_{umass}$ [43,44].

- **$C_{v}$** measure employs a sliding window, a one-set segmentation of top words, and an indirect confirmation measure that employs normalized pointwise mutual information (NPMI) and cosine similarity.
- The **$C_{umass}$** measure is rooted in document co-occurrence counts, a one-preceding segmentation, and a logarithmic conditional probability as a confirmation measure.

Then, the process of applying topic modeling followed these steps:

1. We generated multiple models to determine the optimal number of topics, relying on the previously mentioned coherence measures. After analysis, 12 topics were identified as optimal, achieving a $C_{v}$ score of 0.364 and a $C_{umass}$ score of 0.573.
2. We conducted initial manual topic labeling based on the first five words of each topic.
3. Ten random papers from each topic were reviewed to refine topic delimitation.
4. We assigned the final labels to each topic.

Using the generated topics, we proceeded to evaluate each paper to determine the corresponding topics. It is essential to note that in LDA, documents can be linked to multiple topics with assigned weights. We calculated the proportion of each topic as follows:

(1) $$Proportion\_topic_{j}=\frac{\sum_{i=1}^{N}weight\_topic_{ij}}{N}*100$$

Then, the proportion of topic *j* was computed as the aggregate of the weights assigned to topic *j* across each document from *i* to *N* divided by the total number of documents in the corpus (*N*). Additionally, we computed the proportion of each topic for each individual year. This calculation employed the same formula as presented in Equation (1), with the difference that only the papers corresponding to each specific year were considered.

Furthermore, to discover primary topics based on paper keywords, we applied the same cleaning method previously applied to the full text to the keyword collection. Subsequently, we manually inspected the data to merge similar keywords, consolidating, for instance, terms like “technology-enhanced learning” and “technology enhanced learning” into one. This approach ensures aggregated counts when keywords are slightly different, enhancing the accuracy of our analysis.

### 3.7. Network Analysis

The next stage involved a network analysis using the metadata obtained from the collected papers. Specifically, we built two different networks:

- **A co-authorship network**. This network is an undirected graph that describes collaboration between authors within a collection of documents. Each node within the graph represents an author from the collection, while edges connect authors who have collaboratively worked on one or more papers. Co-authorship is commonly regarded as an indicator of research collaboration, bringing together diverse talents to enhance scientific credibility [45]. In our work, we used the metadata from each paper to build the complete network.
- **A citation network**. This network constitutes a directed graph that captures citations among documents within the collection. Nodes correspond to documents in the collection, and edges are directed from citing documents to the documents they cite. Since citations of others papers are hand-picked by the authors as being related to their research, the citations can be considered to judge relatedness. Usually, direct references or citations are more likely between papers with temporal separation, rather than those published closely in time [46]. In our research, we leveraged the references extracted from papers’ full texts to identify and represent citations. To uniquely identify each paper in the graph, we created an identifier comprising the first author’s name, the initial word of the paper title, and the year of publication (e.g., for a paper authored by “Berns A.”, and published in 2013 with the title “Using a 3D online game to assess students’ foreign language acquisition and communicative competence”, the identifier would be “BernsUsing2013”).

To build both networks, we used two different libraries in Python: *Networkx* and *GephiStreamer*. *Networkx* is a Python package designed for creating, manipulating, and analyzing complex network structures. Its primary function is enabling in-depth graph analysis rather than visualization. Thus, we used *Networkx* to create graphs and calculate some interesting measures, such as centrality or closeness. Since *Networkx* only provides basic visualizations, we wanted to generate a better static visualization for this work. For this visualization purpose, we used *Gephi*, an open-source software package developed for network analysis and visualization using Java on the NetBeans platform. To integrate it with our code, we employed *GephiStreamer*, a Python module that facilitates the direct streaming of graphs into the *Gephi* platform.

### 3.8. Open Data and Analyses

In this final stage, we have ensured the complete reproducibility of our research [36]. To achieve this objective, we have uploaded the complete database of parsed plain texts, metadata associated with each year, and the entire codebase including scripts and notebooks. These scripts offer the capability to replicate our study systematically and obtain the same results we did. Moreover, this also allows other researchers to fully re-apply this methodology in other contexts.

## 4. Results

### 4.1. RQ1: Main Topics of EC-TEL Based on Keywords and Topic Modeling

After implementing the proposed methodology detailed in the previous section, we present a comprehensive summary of each topic, including its name, description, and five associated keywords. This summary can be found in Table 1.

**Figure 3:**
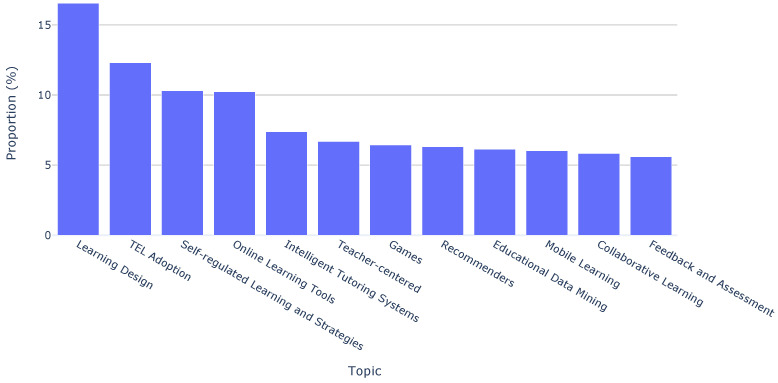
Topic distributions across all papers.

Moreover, we can see the topic distributions across the last ten years of the conference in Figure 3. This visualization illustrates that the most recurrent topics were “Learning Design”, “TEL Adoption”, and “Self-regulated Learning and Strategies”, as opposed to “Collaborative Learning” and “Feedback and Assessment”, which were not so popular.

**Figure 4:**
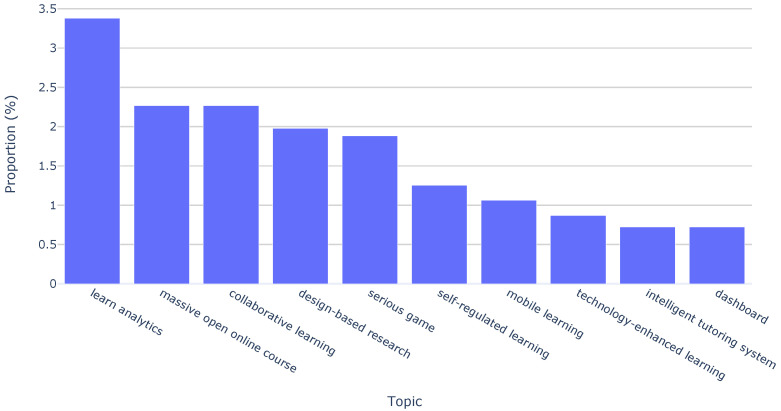
Keyword distribution across all papers.

In addition to this topic-based analysis, we extended our investigation by employing paper keywords. This complementary approach allowed us to determine the distribution of the top ten keywords over the last decade of the EC-TEL. Figure 4 shows that the most frequently recurring keywords are “learn analytics” (3.38%), “massive open online course” (2.27%), and “collaborative learning” (2.27%). Although they are not in the top ten frequent keywords, we have to note the importance of keywords such as “augmented reality” and “virtual reality”. These keywords are directly related to sensor-driven technologies and are present in the collection, highlighting their association with emerging trends in the area.

### 4.2. RQ2: Evolution of Topics across the Previous Ten Years

**Figure 5:**
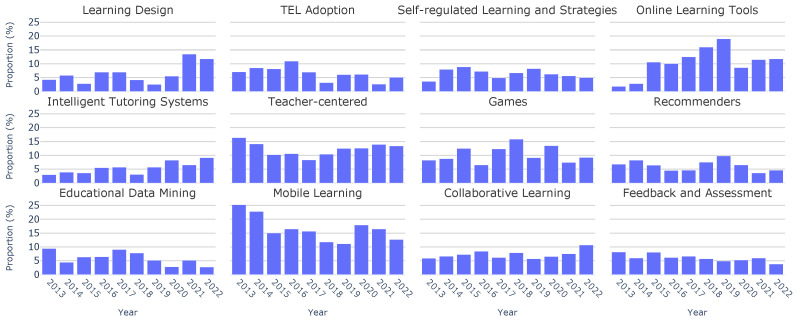
Topic distributions by year.

With our comprehensive analysis of keywords and topics across the entire decade completed, the next step is to perform a year-by-year examination. Figure 5 shows the evolution of topic distributions over the years. Specific topics such as “Learning Design” have gained attention over the years, going from a 4.28% occurrence in 2013 to a substantial peak of 13.49% in 2021. Moreover, topics like “Teacher-centered” have exhibited a consistent frequency throughout the years. Finally, some other topics, such as“Recommenders”, have experienced a gradual decline in popularity over time.

**Figure 6:**
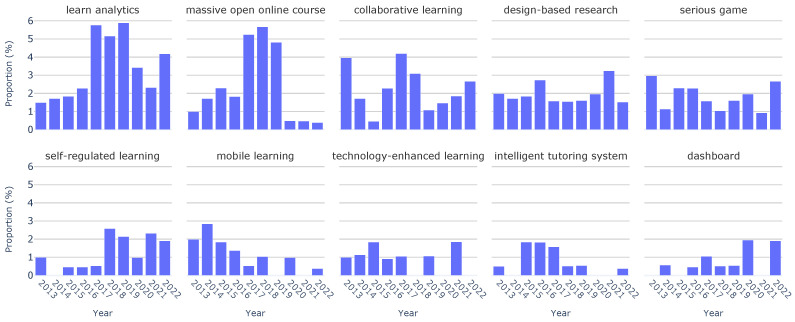
Keyword distribution by year.

Analogously, in Figure 6, we observe the temporal evolution of keyword distributions. We observe that some keywords, such as “design-based research”, reappear year after year, with a consistently stable distribution over the last decade. Furthermore, we note other keywords that have experienced a substantial surge in frequency. This is the case for “learn analytics”, increasing from 1.49% in 2013 to a peak of 5.88% in 2019. Similarly, “massive open online course” increased from 0.99% in 2013 to a maximum of 5.67% in 2018. What we discover looking at Figure 6 is that the majority of keywords registered a decline in frequency from 2020 to 2022. This shift could potentially signify emerging trends in 2022 or a greater diversity of paper topics during this period.

### 4.3. RQ3: Interaction between Papers and Authors

**Figure 7:**
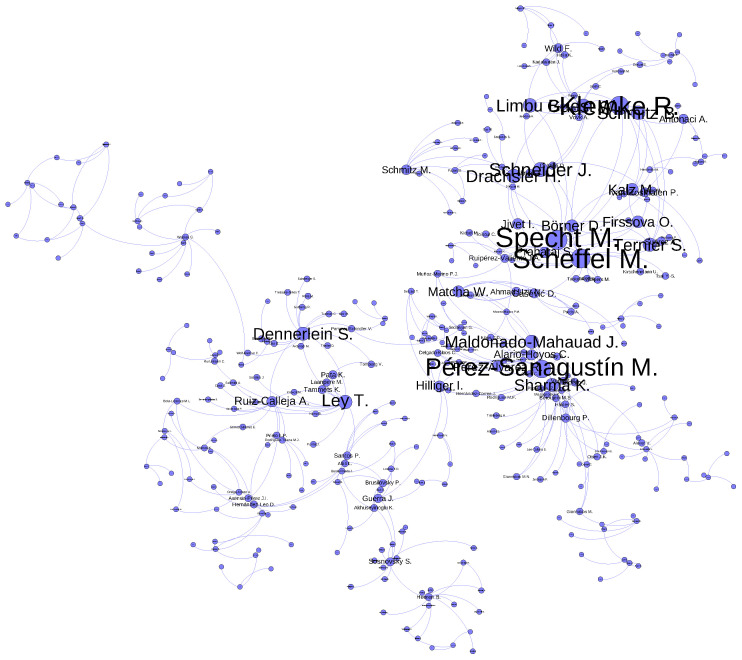
Co-authorship network.

Moving on to Figure 7, we find the co-authorship network built using our code and subsequently streamed into *Gephi*. In this graph, each node represents an author. It is worth nothing that, in our plot, we only represent the giant components (a giant component is a connected component covering a substantial proportion of the entire nodes within the network).

The dataset includes 1331 author names (an average of 3.08 authors per paper), with 948 of those author being names unique. Furthermore, the top five central authors are “Scheffel M.”, “Sharma K.”, “Dennerlein S.”, “Ley T.”, and “Guest W.” These particular authors have a substantial level of collaboration with a large proportion of other authors who also hold central positions within the graph. Furthermore, the authors with a larger amount of papers published are “Specht M.” (2.78%), “Drachsler H.” (2.55%), “Kalz M.” (2.32%), “Pérez-Sanagustín M.” (2.32%), and “Sharma K.” (2.09%).

The giant component shown involves 349 nodes (36.8% of the authors). This implies that the remaining authors, constituting 63.2%, do not engage in collaboration with the authors highlighted in this giant component. This observation points towards the existence of sub-communities within the broader context of the EC-TEL conference. The community structure is characterized by fragmentation and weak interconnections, with an overall density of only 0.007.

**Figure 8:**
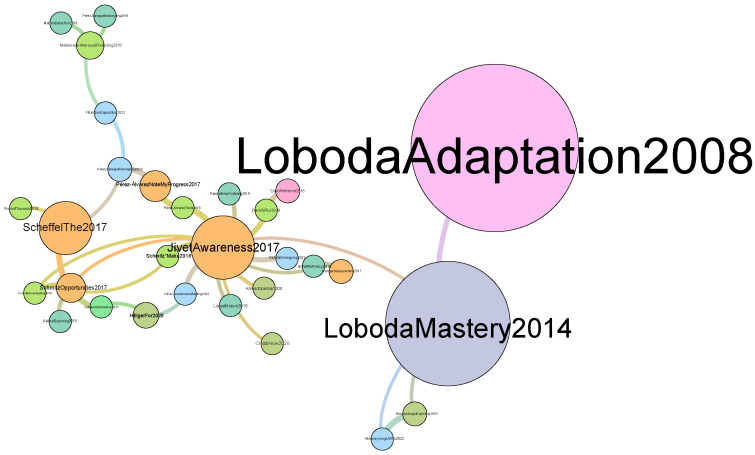
Citation network.

Furthermore, Figure 8 provides a visual representation of the citation network generated through our code and subsequently streamed into *Gephi*. In this visualization, each node corresponds to a paper within the network, while the color scheme represents the various publication years. Note that, in our plot, only the giant component is shown.

The framework has found 173 references between papers. The top five central papers are “Awareness is not enough: Pitfalls of learning analytics dashboards in the educational practice” [47], “Opportunities and challenges in using learning analytics in learning design” [48], “Digital didactical designs of learning expeditions” [49], “Mastery grids: An open source social educational progress visualization” [50], and “Learning analytics for professional and workplace learning: A literature review” [51]. Looking at the top ten central papers, we note that one of them was published in 2007, one of them in 2008, one in 2013, three in 2014, three in 2017, and one in 2018. Since these are the papers that have been most cited between them, we note that they are the ones having a major influence on the community of the EC-TEL.

## 5. Discussion

Our study automatically found the EC-TEL’s main trends during the last ten years, basing our analysis on two primary sources: full manuscripts and keywords. We can see a summary of our research findings in Table 2 and Table 3. It is evident that a substantial portion of the identified topics is shared across both methods, such as games, mobile learning, design, and collaborative learning, reaffirming our results’ validity.

However, the topics’ distribution and evolution over time are different in most cases when we compare our two approaches. For instance, if we take a look at our analysis using the full texts, the topic “Games” represents 6.43% of the papers, meanwhile its distribution decreases to 1.88% when relying solely on paper keywords. This can be explained by the fact that keywords are often meticulously chosen by authors to align with current research paradigms and communities, while the LDA model extracts latent topics from the full content of articles, potentially presenting a more authentic reflective picture of the paper’s actual theme.

Moreover, it is crucial to consider the role of sensors in shaping the future of the Edtech area. Although we noted that sensor-related terms like AR/VR and “Mobile learning” have gained attention in recent years, current trends in the area do not explicitly address the broader potential of sensor-driven technologies. This presents an opportunity for further exploration and innovation. Integrating sensor technologies, such as wearable devices, environmental sensors, or physiological sensors, can help us to leverage the learning experience by capturing rich data, enabling personalized feedback, and facilitating adaptive learning environments. For example, sensor-driven technologies can capture real-time data on student interactions, engagement, and performance. These data could be analyzed to gain insights into student behavior and potential difficulties, enabling educators to personalize their classes. Another example is the use of physiological sensors that can monitor students’ stress levels, concentration, and well-being. This information can be used to optimize learning and support the mental and physical health of learners. Moreover, the use of wearable devices and sensors can enhance accessibility and inclusion by supporting students with difficulties, providing personalized support and accommodations. Specifically, the IoT has brought a new era of connected devices and systems, but the reliability and security of these systems are critical concerns that must be addressed [52]. Formal methods can assist in verifying the correctness of algorithms and analytics applied to IoT data, which is critical for decision making [53]. Combining the IoT and formal methods in EdTech can lead to robust and reliable sensor-driven technologies. Future research should investigate the incorporation of these sensor-driven approaches within the context of Edtech, as this holds the promise of unlocking new possibilities for enhancing teaching and learning outcomes.

Furthermore, while our study found the most central authors and papers, it also found authors that have published a large amount of papers during the last ten years. If we take a look at our results, we see that publishing a larger amount of papers does not imply being a more central author, as the centrality measure depends on the relationship with other authors. In fact, only one author (“Sharma K.”) appears in both lists (most central authors and authors with a larger amount of papers at the conference). If we look at the citation network, we see that two of the biggest nodes are papers by “Loboda”. However, we do not see this author highlighted in the co-authorship network. In addition, we also see that almost all authors that we highlighted in our results appear as the main author of the paper in the citation network. This observation aligns with the ongoing discourse on the balance between the quality and quantity of scientific publications. In this setting, researchers have been gradually shifting their focus from sheer quantity to prioritizing the quality of research [54,55].

Comparing our work to other studies in terms of methodology reveals interesting insights. For instance, Choi et al. [56] analyzed research trends in personal information privacy. In their study, they employed text mining on the abstract of each retrieved paper, determining the optimal number of topics by maximizing the log-likelihood of the topic model. In contrast, our research employs two coherence measures to determine the suitable number of topics. Unlike likelihood measures based on held-out data, these coherence measures exhibit a stronger correlation with human topic ranking data, which is considered the gold standard for evaluating topic interpretability [20]. We can also see similar research in [24], where the authors analyzed the first five years of EC-TEL proceedings. Unlike our research (which uses full-text manuscripts), this paper focused on the analysis of metadata, including authors and citations. This work found that the most cited paper was published by Stefaner et al. in 2007 [33]. In our research, this paper is the ninth most central paper in the collection, but it does not appear in the giant component shown in the citation graph. This finding also reveals a very fragmented community that is only weakly connected, and this is also confirmed in our analysis. In Figure 7, only the giant component in shown, and only 36.8% of authors are visible in the network, meaning that there is a large proportion of nodes that is not connected.

As previously indicated in Section 2, earlier research has explored topic trends within this domain. For instance, in their study, Chen et al. [22] employed LDA topic finding using paper abstracts to discover trends. Several of their identified topics, such as “Collaborative learning” and “Feedback”, align with our findings. However, they also highlighted topic less common today, such as ”Blended learning”. Likewise, Hung [23] conducted an investigation on e-learning trends from 2000 to 2008. Employing text mining, they clustered documents based on abstract similarities and presented these clusters in a hierarchical tree structure. This study revealed some more specific trends such as “Architecture and standards”, “Simulations”, and “E-learning applications in medical education and training”. Our research also aligns with some of these trends. For instance, the trend labeled “community and interactions” corresponds with our topic “Collaborative learning”. The emergence of novel trends like “dashboards” is promising, offering the potential to extract more nuanced insights from existing data, presenting a contemporary challenge to address.

Finally, we would like to discuss into the implications of our findings for educational practice and policy. First, the identified trends and networks can help guide future research directions, ensuring that academic research focuses on the practical challenges and needs of the EdTech area. Second, the identification of collaborative networks could help to encourage partnerships between institutions, researchers, and organizations, facilitating the exchange of knowledge and resources between them. Moreover, understanding the latest trends in EdTech research can guide the integration of new technologies and pedagogical approaches in the classrooms. Finally, policy makers can use the findings of our study to develop policies that support the growth of the area by ensuring that regulations and initiatives align with the evolving EdTech environment.

## 6. Conclusions and Future Work

This study aims to discover the main EdTech trends in the last ten years by analyzing papers from the EC-TEL conference using an “NLP-enhanced bibliometrics” approach. This methodology combines NLP, topic modeling, and social network analysis instead of classic qualitative reviews that require much more time to be performed. Furthermore, this is an open science methodology and easily reusable in many other contexts. We have made the code and dataset accessible publicly so that other researchers can reproduce our work in any context (including ours). Some of the most frequent topics that we found are “Learning Design”, “TEL Adoption”, and “Learning Analytics”, which have gained popularity over time. Other topics, like “Online Learning Tools”, have also been very popular, but their popularity has been decreasing over time. However, it is important to note that our analysis reveals a lack of focus on sensor-driven technologies in the area, apart from AR/VR and mobile learning.

This work has some limitations. First of all, we did not use the complete proceedings of the EC-TEL, as we excluded demo and poster papers from our analysis. It is worth noting that including demo and poster papers could be interesting but could also introduce some bias due to the papers’ reduced sizes. Our keyword analysis also faces limitations as it relies on authors’ keywords, assuming they comprehensively include all addressed topics within each paper, but this is not always the case in practice. In regard to the LDA algorithm, while we carefully selected the number of topics following our methodology, there remains the constraint of this choice, since there might be some hidden topics that we have not discovered. Another limitation is related to the fact that some authors and organizations present names in different ways, which can skew our network analysis results by introducing bias. Furthermore, it is crucial to acknowledge that this trend- and topic-finding methodology may not provide the same level of detail as more qualitative and manual reviews. Nevertheless, it does provide valuable and sufficient information, particularly within the area of bibliometrics.

Given that our methodology provides a novel approach to perform quick bibliometric reviews, as part of our future work, we will focus on developing and validating a framework that automates this process, allowing rapid and automatic results generation using any corpus of papers. As the role of sensors in education becomes increasingly prominent, their impact on teaching and learning needs to addressed. Therefore, future work should aim to integrate sensor-based data analysis and explore how sensors can enhance educational experiences in the area. Since there is existing research using sensor-driven technologies in the e-learning field, we would like to extend our analysis to a broader range of areas, exploring publications from various conferences and journals in the e-learning domain. Moreover, as this conference is part of the International Alliance to Advance Learning in the Digital Era (IAALDE), we would like to perform a deeper analysis using a larger amount of societies that are part of this alliance, which would allow for more general and interesting results on the EdTech area. Additionally, we plan to extend the network analysis built by developing deeper networks with more advanced network measures. To ascertain the efficacy of our methodology, we would like to conduct a comparison between the results obtained by an existing review conducted using classic methodologies in bibliographic analysis and the ones obtained using our approach. We expect our study to help overcome the limitations of qualitative analyses in EdTech by using NLP to process large amounts of research objectively.

## Figures and Tables

**Table 1:** Summary of each of the detected topics.

Topic	Description	Main Terms
Learning Design	Papers focusing on the learning design to ensure the quality of instruction and design-based research	Learn, design, activity, tool, process
Technology- Enhanced Learning (TEL) Adoption	Papers with the objective of exploring the factors and challenges associated with the integration and utilization of technology in educational settings	Learn, education, barrier, perception, survey
Self-regulated Learning and Strategies	Studies where the students monitor their performance and reflect on it, using this reflection to adjust and improve upcoming tasks	Task, error, test, feedback, strategy
Online Learning Tools	Research about online learning tools (e.g., online courses, massive open online courses)	Activity, student, video, time, learner
Intelligent Tutoring Systems	Includes papers that aim to provide immediate and customized instruction or feedback to learners and customizing those learning experiences	Student, test, tutor, technology, error, skill
Teacher-centered	Providing approaches that are centered on teachers, who are actively involved in the learning process	Teacher, student, classroom, activity, lesson
Games	Includes papers that aim to use games and gamification to improve learning	Game, child, player, scenario, gamification
Recommenders	Papers that use recommender systems applied in education, along with papers aiming to make research accessible (open education)	Resource, tag, user, learn, recommend
Educational Data Mining (EDM)	Focused on applying data mining, machine learning and/or statistics techniques to information generated from educational environments	Model, item, performance, measure, training
Mobile Learning	Research to improve training and education by incorporating portable devices (e.g., smartphones, tablets)	Application, device, user, experience, learn
Collaborative Learning	Research that promotes the use of groups to enhance learning through working together	Group, collaboration, knowledge, member, social
Feedback and Assessment	Research that investigates the effectiveness and impact of feedback mechanisms and assessment practices in educational contexts	Feedback, question, argument, assessment, student

**Table 2:** Topic analysis results summary. Upward arrows indicate an increase in the proportion of articles on that particular topic over the specified time period. Downward arrows indicate a decrease in the proportion, while rightward arrows indicate a relatively stable proportion of articles.

Method	Topic	General Proportion (RQ1)	Evolution 2013–2022 (RQ2)
**Topic finding**	Learning Design	16.5%	↑ 4.3–11.8%
	TEL Adoption	12.3%	↓ 7.0–5.1%
	Self-regulated Learning and Strategies	10.3%	→ 3.6–5.0%
	Online Learning Tools	10.2%	↑ 1.8–11.8%
	Intelligent Tutoring Systems	7.4%	↑ 3.0–9.2%
	Teacher-centered	6.7%	→ 16.3–13.3%
	Games	6.4%	→ 8.2–9.2%
	Recommenders	6.3%	↓ 6.8–4.6%
	EDM	6.1%	↓ 9.4–2.7%
	Mobile Learning	6.0%	↓ 25.3–12.7%
	Collaborative Learning	5.8%	↑ 5.9–10.7%
	Feedback and Assessment	5.6%	↓ 8.2–3.8%
**Keyword analysis**	learning analytics	3.4%	↑ 1.5–4.2%
	massive open online course	2.3%	↓ 1.0–0.4%
	collaborative learning	2.3%	↓ 4.0–2.7%
	design-based research	2.0%	→ 2.0–1.5%
	serious game	1.9%	→ 3.0–2.7%
	self-regulated learning	1.3%	↑ 1.0–1.9%
	mobile learning	1.1%	↓ 2.0–0.4%
	technology-enhanced learning	0.9%	↓ 1.0–0.0%
	intelligent tutoring system	0.7%	→ 0.5–0.4%
	dashboard	0.7%	↑ 0.0–1.9%

**Table 3:** Network analysis results summary.

Method	Finding	Results (RQ3)
**Co-authorship network**	Number of authors	1331 (948 unique)
	Central authors	“Scheffel M.”, “Sharma K.”, “Dennerlein S.”, “Ley T.”, and “Guest W.”
	Most frequent authors	“Specht M.”, “Drachsler H.”, “Kalz M.”, “Pérez-Sanagustín M.”, and “Sharma K.”,
**Citation network**	References between papers	143
	Central papers	[47,48,49,51]

## Data Availability

All the data used in this study are available at the following Open Science Framework (OSF) repository: https://osf.io/jtnaq/?view_only=3d4974a06fc441e88cc2d7b5fc51a925 (accessed on 29 August 2023).

## References

[1] Kirkwood A., Price L. (2014). Technology-enhanced learning and teaching in higher education: What is ‘enhanced’and how do we know? A critical literature review. Learn. Media Technol..

[2] Fabry D.L., Higgs J.R. (1997). Barriers to the effective use of technology in education: Current status. J. Educ. Comput. Res..

[3] Noble H., Smith J. (2018). Reviewing the Literature: Choosing a Review Design. Evid. Based Nurs..

[4] Rickinson M., May H. (2009). A Comparative Study of Methodological Approaches to Reviewing Literature.

[5] Kovačević A., Konjović Z., Milosavljević B., Nenadic G. (2012). Mining methodologies from NLP publications: A case study in automatic terminology recognition. Comput. Speech Lang..

[6] Liddy E.D. (2001). Natural language processing. Encyclopedia of Library and Information Science.

[7] Pritchard A. (1969). Statistical bibliography or bibliometrics. J. Doc..

[8] Kanna S., von Rosenberg W., Goverdovsky V., Constantinides A.G., Mandic D.P. (2018). Bringing wearable sensors into the classroom: A participatory approach [SP education]. IEEE Signal Process. Mag..

[9] Cowling M.A., Crawford J., Vallis C., Middleton R., Sim K.N. (2022). The EdTech difference: Digitalisation, digital pedagogy, and technology enhanced learning. J. Univ. Teach. Learn. Pract..

[10] Rehman Z.u. (2023). Trends and Challenges of Technology-Enhanced Learning in Geotechnical Engineering Education. Sustainability.

[11] Ramlowat D.D., Pattanayak B.K. (2019). Exploring the internet of things (IoT) in education: A review. Proceedings of the Information Systems Design and Intelligent Applications: Proceedings of Fifth International Conference INDIA, 2018.

[12] Burchfield R.W. (1972). A Supplement to the Oxford English Dictionary.

[13] Broadus R.N. (1987). Toward a definition of “bibliometrics”. Scientometrics.

[14] Diodato V.P., Gellatly P. (2013). Dictionary of Bibliometrics.

[15] Gao Y., Ge L., Shi S., Sun Y., Liu M., Wang B., Shang Y., Wu J., Tian J. (2019). Global trends and future prospects of e-waste research: A bibliometric analysis. Environ. Sci. Pollut. Res..

[16] Lv P.H., Wang G.F., Wan Y., Liu J., Liu Q., Ma F.C. (2011). Bibliometric trend analysis on global graphene research. Scientometrics.

[17] Miau S., Yang J.M. (2018). Bibliometrics-based evaluation of the Blockchain research trend: 2008–March 2017. Technol. Anal. Strateg. Manag..

[18] Chowdhury G.G. (2003). Natural language processing. Annu. Rev. Inf. Sci. Technol..

[19] Buitelaar P., Bordea G., Coughlan B. Hot topics and schisms in NLP: Community and trend analysis with saffron on ACL and LREC proceedings. Proceedings of the 9th Edition of Language Resources and Evaluation Conference (LREC2014).

[20] Syed S., Spruit M. Full-text or abstract? examining topic coherence scores using latent dirichlet allocation. Proceedings of the 2017 IEEE International Conference on Data Science and Advanced Analytics (DSAA).

[21] Abu-Jbara A., Ezra J., Radev D. Purpose and polarity of citation: Towards nlp-based bibliometrics. Proceedings of the 2013 Conference of the North American Chapter of the Association for Computational Linguistics: Human Language Technologies.

[22] Chen X., Zou D., Xie H. (2020). Fifty years of British Journal of Educational Technology: A topic modeling based bibliometric perspective. Br. J. Educ. Technol..

[23] Hung J.l. (2012). Trends of e-learning research from 2000 to 2008: Use of text mining and bibliometrics. Br. J. Educ. Technol..

[24] Reinhardt W., Meier C., Drachsler H., Sloep P. Analyzing 5 years of EC-TEL proceedings. Proceedings of the European Conference on Technology Enhanced Learning.

[25] Li X., Lei L. (2021). A bibliometric analysis of topic modelling studies (2000–2017). J. Inf. Sci..

[26] Urru S., Sciannameo V., Lanera C., Salaris S., Gregori D., Berchialla P. (2022). A topic trend analysis on COVID-19 literature. Digit. Health.

[27] Chen X., Zou D., Xie H. (2022). A decade of learning analytics: Structural topic modeling based bibliometric analysis. Educ. Inf. Technol..

[28] Gurcan F., Ozyurt O., Cagitay N.E. (2021). Investigation of emerging trends in the e-learning field using latent dirichlet allocation. Int. Rev. Res. Open Distrib. Learn..

[29] Pramanik P., Jana R.K. (2022). Identifying research trends of machine learning in business: A topic modeling approach. Meas. Bus. Excell..

[30] Liu G., Nzige J.H., Li K. (2019). Trending topics and themes in offsite construction (OSC) research: The application of topic modelling. Constr. Innov..

[31] Scott J. (1988). Social network analysis. Sociology.

[32] Otte E., Rousseau R. (2002). Social network analysis: A powerful strategy, also for the information sciences. J. Inf. Sci..

[33] Stefaner M., Dalla Vecchia E., Condotta M., Wolpers M., Specht M., Apelt S., Duval E. MACE–enriching architectural learning objects for experience multiplication. Proceedings of the European Conference on Technology Enhanced Learning.

[34] Atanassova I., Bertin M., Mayr P. (2019). Mining scientific papers: NLP-enhanced bibliometrics. Front. Res. Metrics Anal..

[35] Gomez M.J., Ruipérez-Valiente J.A., Clemente F.J.G. (2021). Bibliometric Analysis of the Last Ten Years of the European Conference on Technology-Enhanced Learning. Proceedings of the European Conference on Technology Enhanced Learning.

[36] Gomez M.J. Supplementary Materials: A NLP-Enhanced Bibliometrics Study of the Technology-Enhanced Learning Field, 2023. https://osf.io/jtnaq/?view_only=3d4974a06fc441e88cc2d7b5fc51a925.

[37] EATEL. Proceedings of the EC-TEL Conference.

[38] (2021). Responsive and Sustainable Educational Futures. Proceedings of the 18th European Conference on Technology Enhanced Learning.

[39] Elsevier. About Scopus, 2021.

[40] Science W.O. Web of Science, 2021. https://clarivate.com/products/scientific-and-academic-research/research-discovery-and-workflow-solutions/webofscience-platform/.

[41] Yao L., Mimno D., McCallum A. Efficient methods for topic model inference on streaming document collections. Proceedings of the 15th ACM SIGKDD International Conference on Knowledge Discovery and Data Mining.

[42] O’callaghan D., Greene D., Carthy J., Cunningham P. (2015). An analysis of the coherence of descriptors in topic modeling. Expert Syst. Appl..

[43] Röder M., Both A., Hinneburg A. Exploring the space of topic coherence measures. Proceedings of the Eighth ACM International Conference on Web Search and Data Mining.

[44] Kapadia S. (2019). Evaluate Topic Models: Latent Dirichlet Allocation (LDA). https://towardsdatascience.com/end-to-end-topic-modeling-in-python-latent-dirichlet-allocation-lda-35ce4ed6b3e0.

[45] Kumar S. (2015). Co-authorship networks: A review of the literature. Aslib J. Inf. Manag..

[46] Lu W., Janssen J., Milios E., Japkowicz N., Zhang Y. (2007). Node similarity in the citation graph. Knowl. Inf. Syst..

[47] Jivet I., Scheffel M., Drachsler H., Specht M. Awareness is not enough: Pitfalls of learning analytics dashboards in the educational practice. Proceedings of the European Conference on Technology Enhanced Learning.

[48] Schmitz M., Van Limbeek E., Greller W., Sloep P., Drachsler H. (2017). Opportunities and challenges in using learning analytics in learning design. Proceedings of the Data Driven Approaches in Digital Education: 12th European Conference on Technology Enhanced Learning, EC-TEL 2017.

[49] Jahnke I., Norqvist L., Olsson A. Digital didactical designs of learning expeditions. Proceedings of the European Conference on Technology Enhanced Learning.

[50] Loboda T.D., Guerra J., Hosseini R., Brusilovsky P. Mastery grids: An open source social educational progress visualization. Proceedings of the European Conference on Technology Enhanced Learning.

[51] Ruiz-Calleja A., Prieto L.P., Ley T., Rodríguez-Triana M.J., Dennerlein S. (2017). Learning analytics for professional and workplace learning: A literature review. Proceedings of the European Conference on Technology Enhanced Learning.

[52] Krichen M. (2023). A Survey on Formal Verification and Validation Techniques for Internet of Things. Appl. Sci..

[53] Hofer-Schmitz K., Stojanović B. (2020). Towards formal verification of IoT protocols: A Review. Comput. Netw..

[54] Fischer J., Ritchie E.G., Hanspach J. (2012). Academia’s obsession with quantity. Trends Ecol. Evol..

[55] Donaldson M.R., Cooke S.J. (2014). Scientific publications: Moving beyond quality and quantity toward influence. BioScience.

[56] Choi H.S., Lee W.S., Sohn S.Y. (2017). Analyzing research trends in personal information privacy using topic modeling. Comput. Secur..

